# DNA Barcoding for the Identification and Authentication of Animal Species in Traditional Medicine

**DOI:** 10.1155/2018/5160254

**Published:** 2018-04-22

**Authors:** Fan Yang, Fei Ding, Hong Chen, Mingqi He, Shixin Zhu, Xin Ma, Li Jiang, Haifeng Li

**Affiliations:** ^1^Institute of Forensic Science, Ministry of Public Security, Beijing 100038, China; ^2^Beijing Engineering Research Center of Crime Scene Evidence Examination, Institute of Forensic Science, Beijing 100038, China; ^3^Center for Bioresources & Drug Discovery and School of Biosciences & Biopharmaceutics, Guangdong Pharmaceutical University, Guangzhou, Guangdong 510006, China

## Abstract

Animal-based traditional medicine not only plays a significant role in therapeutic practices worldwide but also provides a potential compound library for drug discovery. However, persistent hunting and illegal trade markedly threaten numerous medicinal animal species, and increasing demand further provokes the emergence of various adulterants. As the conventional methods are difficult and time-consuming to detect processed products or identify animal species with similar morphology, developing novel authentication methods for animal-based traditional medicine represents an urgent need. During the last decade, DNA barcoding offers an accurate and efficient strategy that can identify existing species and discover unknown species via analysis of sequence variation in a standardized region of DNA. Recent studies have shown that DNA barcoding as well as minibarcoding and metabarcoding is capable of identifying animal species and discriminating the authentics from the adulterants in various types of traditional medicines, including raw materials, processed products, and complex preparations. These techniques can also be used to detect the unlabelled and threatened animal species in traditional medicine. Here, we review the recent progress of DNA barcoding for the identification and authentication of animal species used in traditional medicine, which provides a reference for quality control and trade supervision of animal-based traditional medicine.

## 1. Introduction

Traditional medicine (TM) has been widely used for the prevention and treatment of various common ailments and complicated illnesses in human history, and the use of animals as medicine, which is known as zootherapy, has long been an essential part of traditional therapeutic practices, such as Traditional Chinese Medicine (TCM), Kampo medicine, Ayurvedic medicine, and American folk medicine [1–6]. Besides the traditional usages, modern clinical studies have demonstrated that animal-based TMs possess a number of pharmacological effects, including anti-inflammatory, antitumor, anti-infective, anticonvulsant, analgesic, and immunomodulatory activities [6–9]. Therefore, zootherapy continues to serve as an important complementary and alternative therapy in modern societies. For example, over 1,500 animal species have medicinal benefits according to the historical records in China, and approximately 77 kinds of medicinal animals and 50 kinds of medicinal materials derived from animal sources have been included in the Chinese Pharmacopoeia 2015 Edition [1, 10]. In Brazil and Latin America, at least 354 and 584 animal species have been reported to be used in TM, respectively [4, 5]. It is estimated that animal-derived TM has been increasingly used in many countries and its annual global trade accounts for billions of dollars [2]. Moreover, recent studies have revealed that animal-based TM contains a variety of bioactive compounds, which provides a valuable chemical library for drug discovery [11], indicating the potential of medicinal animals in developing modern pharmaceuticals.

During recent centuries, a wide range of wild animal species is known to be threatened with extinction; for example, according to the latest statistics of the International Union for Conservation of Nature (IUCN) Red List of Threatened Species, the number of endangered and critically endangered animal species has reached 7597 and 5101, respectively [12]. Such rapid loss of biodiversity is closely associated with animal habitat loss and human overexploitation. For instance, despite some parts such as horns and secretions, almost all types of tissues and organs used in TM require animal sacrifice, which inflicts substantial stress to medicinal animal resources [2, 13, 14]. To solve the species crisis, the trade of threatened animals has been regulated under international legislation including the Convention on International Trade in Endangered Species (CITES), and the therapeutic application of such species has been forbidden in many countries. However, some countries such as those in East and Southeast Asia still utilize threatened animals for medicinal purposes [2, 15], and the illegal trade of medicinal animals further impairs biodiversity [16, 17]. Moreover, a variety of adulterants and counterfeits has emerged in the market for greater interests, which brings challenges to the safety of animal-based TM. Therefore, identification and authentication of animal species is of vital importance for TM trade supervision and quality control.

It is known that animal-based TMs are mainly derived from animal tissues, organs, or metabolites, and these parts are usually processed into diverse preparations such as slice, powder, and tablet, resulting in difficulties for morphology-based species identification. In addition, several commercial technologies used for species identification, including chromatography and immunoassay, are relatively costly and expertise-dependent, which is not suitable for bulk detection of TM preparations and forensic specimens [18, 19]. Therefore, developing an accurate and efficient identification method represents an urgent need for animal-based TM. In recent decade, advance in molecular techniques has promoted the application of simple and precise DNA analysis in taxonomic field. Among the prevailing genome-based approaches, DNA barcoding provides a robust strategy to identify existing species and discover unknown species via comparative analysis of sequence variation [19–21]. Extensive studies have shown that DNA barcoding is capable of identifying a wide range of animal species, including mammals, birds, reptiles, amphibians, fishes, and crustaceans [21–24]. Moreover, many international organizations such as the Consortium for the Barcode of Life (CBOL) and the Barcode of Life Data System (BOLD) have been established to promote DNA barcoding as a global standard for species identification. In addition to taxonomy, DNA barcoding has recently been applied in various fields, including medicine and food science, forensics, and conservation biology [19, 20, 25–27]. In the field of TM, DNA barcoding has been widely used to authenticate herbal sources [19, 20]. However, studies on identifying medicinal animal species including the threatened species and discriminating the authentic TMs from the adulterants are relatively less addressed. Therefore, we tend to review the general process and current progress of DNA barcoding in analyzing animal-based TM and then discuss its limitations and potential strategies.

## 2. Animal Identification by DNA Barcoding

### 2.1. DNA Barcoding and Minibarcoding

The accurate species identification by DNA barcoding relies on a suitable DNA barcode, which refers to a standardized sequence (usually less than 1,000 bp) of the genome [21]. The barcode should have universality so that it can be easily amplified from diverse species, and it should contain few insertions or deletions to facilitate sequence alignment. Also, its mutation rate must be sufficient to generate a barcoding gap, which means the maximum intraspecific variation is less than the minimum interspecific distance [21]. For animal identification, the most broadly used barcode marker is mitochondrial cytochrome c oxidase subunit I (COI), which is highly conserved across species employing oxidative phosphorylation for metabolism [22]. Numerous studies have shown that COI-based DNA barcoding can delimit diverse animal species, indicating the high rates of sequence change at species level and constraints on intraspecific divergence in COI sequence [21–24]. For analyzing fresh and well-preserved animal tissues, a full-length barcode such as a 658-bp region of COI gene is recommended as its PCR amplification and sequencing can be feasible. However, animal tissues are usually processed before used as TM materials, and some processing approaches, such as sun-drying, stir-frying, and boiling, can cause DNA degradation, leading to difficulties for PCR amplification of full-length barcodes [19]. Since the amplification feasibility improves with reduced sequence length, a minibarcoding method, which utilizes a shorter region within the standard barcode, has received increasing attention [28]. Recent studies have shown that an array of minibarcodes can be effectively amplified and sequenced from various processed products including TMs and foods and provide sufficient sequence information necessary for species identification [26, 29, 30].

### 2.2. DNA Metabarcoding

It is known that many TM preparations are composed of multiple materials derived from diverse animal and/or plant species. In such mixtures, multiple barcode sequences will be coamplified and the conventional sequencing will generate multiple or overlaying sequencing peaks, resulting in ambiguity or false sequence information [31]. Therefore, DNA metabarcoding has been proposed to identify multiple species within complex samples using next-generation sequencing (NGS) technology [32, 33]. NGS can rapidly yield millions of DNA reads and obtain all representative sequences presented in mixtures, which facilitates a high-throughput multitaxa identification. This technology can also be used for sequencing minibarcodes (approximately 50–400 bp), which makes it suitable for analyzing processed samples with degraded DNA [19]. For example, Arulandhu et al. [34] have shown that a multilocus DNA metabarcoding, including a set of full-length barcodes and minibarcodes combined with Illumina MiSeq amplicon sequencing, can reproducibly identify animal and plant species present in mixtures with both low and high complexity at 1% dry weight content, demonstrating its accuracy and sensitivity in analyzing complex samples. Due to these advantages, DNA metabarcoding has been widely applied in biodiversity research, ecological management, and community analysis [33, 35]. Recently, it has also been used to identify animal species in medicinal preparations, foods, and forensic specimens [34, 36–38]. For instance, Coghlan et al. [36] used mitochondrial 16S ribosomal RNA (16S rRNA) barcode and NGS technology to detect species in 15 complex TCMs presented in the form of powders, capsules, and tablets and revealed that some of them contained CITES-listed animal species and unlabeled species, demonstrating the capability of DNA metabarcoding in analyzing TM formulae.

## 3. General Process of Animal Identification by DNA Barcoding

**Figure 1:**
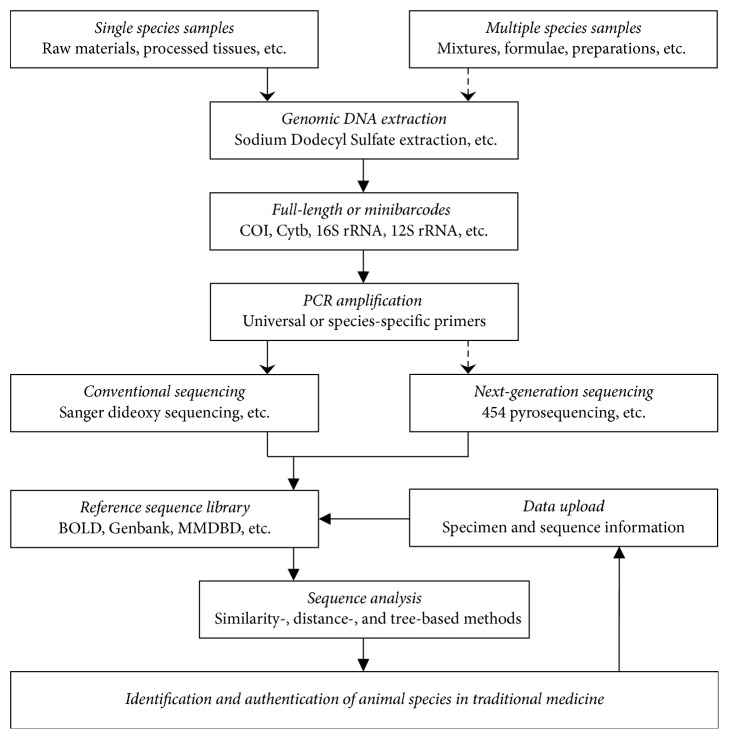
A diagrammatic process of DNA barcoding as well as minibarcoding and metabarcoding for identifying animal species in traditional medicine samples containing single or multiple species.

Although diverse animal tissues and animal-based TMs are used in different studies, the main procedures of DNA barcoding are similar. After extraction of DNA from the tested samples, appropriate DNA barcodes must be selected and then amplified via the polymerase chain reaction (PCR). The amplified regions can be sequenced by conventional methods or NGS according to the sample complexity and then matched to existing sequences from reference database or voucher specimens. Further comparative analysis can be performed to detect the intraspecific and interspecific sequence divergences (Figure 1).

### 3.1. DNA Extraction

Extraction of DNA with high yield and quality is a crucial premise for DNA barcoding of animal species. For some types of animal tissues that contain a small amount of DNA, appropriate sampling and sufficient homogenization are important before DNA extraction. The extraction protocol usually involves several critical steps, including tissue lysis, removal of impurities from DNA, and DNA precipitation [39]. There are many classical methods for extracting out DNA from animal tissues, such as Sodium Dodecyl Sulfate (SDS) extraction, guanidinium thiocyanate-phenol-chloroform extraction, silica matrix-based purification, and magnetic bead-based purification [40]. In many laboratories, these conventional methods have been modified to improve DNA extraction efficiency; for example, Ivanova et al. [41] developed an inexpensive and automation-friendly animal DNA extraction protocol, which employed SDS and proteinase K for tissue lysis followed by silica-based purification using glass fiber filtration plates. In addition, most of these protocols have been developed into commercial kits, which provide standard DNA extraction means for different studies. As different types of animal tissues have distinct characteristics, it is necessary to select appropriate extraction methods or commercial kits (Table 1).

### 3.2. Selection of Barcode Region

Extensive studies have demonstrated the capability of COI region in animal taxonomy; however, this barcode still has several limitations [38, 42]. For instance, COI barcoding region has been found to offer insufficient and unreliable discrimination for some species in the class Gastropod and Anthozoa [43, 44]. In fact, several other genes, such as mitochondrial cytochrome b (Cytb), 16S rRNA, 12S ribosomal RNA (12S rRNA), and nuclear ribosomal internal transcribed spacer (ITS), have also been used for barcoding of animal species [42, 45, 46]. For example, the ITS2 region can achieve an identification rate of 91.7% at the species level among 12,221 kinds of animals recorded in GenBank and differentiate some animal species such as the Argasidae that can be not identified by COI barcode [45]. On the other hand, minibarcodes usually exhibit higher success rate of amplification than full-length barcodes in highly processed samples. However, since the success of taxonomic differentiation is positively correlated with the barcode length, the minibarcode length is usually kept above 100 bp. For example, an approximately 250-bp region of 16S rRNA can be successfully amplified from various medicinal preparations and food products and further provides correct identification of animal species [34, 36, 47]. In addition, as a single DNA barcode may generate insufficient or false identification of hybrid species as well as animal species with high diversity [48], multilocus barcodes can be used to improve the identification accuracy and sensitivity. The DNA barcoding guideline for molecular identification of TCM, which is included in the Appendix of Chinese Pharmacopoeia, has proposed a comprehensive animal identification using both COI and ITS2 barcodes [10].

### 3.3. PCR Amplification of Barcode Region

The amplification efficiency of the barcode region is closely associated with the primer pairs; for example, appropriate barcode primers should be versatile across a wide range of animal species and have high affinity to DNA templates and a balanced melting temperature [49]. Ivanova et al. [50] have developed universal primer sets for amplifying COI barcode. When the universal primers are not applicable for certain taxa or specimens, it is necessary to redesign primers, such as those for minibarcodes [19, 47]. Many molecular biology software and related websites, including Primer Premier, Oligo, and Whitehead, can be used to design and evaluate primers. In addition to the primers, PCR reaction system also contains other necessities including heat-stable DNA polymerases, dNTP mixtures, and DNA templates. The reaction parameters, such as the temperature and time of melting and annealing processes, play an important role in the selective amplification of target templates, and these parameters should be optimized according to the specific circumstances [51].

### 3.4. Sequencing of Amplified Region

As a widely used DNA sequencing method, Sanger dideoxy sequencing is capable of generating sequencing reads up to 1,000 bp [52]. However, Sanger sequencing is low throughput, which makes it suitable for DNA barcoding of single species at a small scale [31, 53]. In contrast, NGS technology can parallelly sequencing multiple DNA fragments in a single reaction [38]. The 454 pyrosequencing is the first commercially available NGS and has been used to analyze various types of mixtures, including environmental specimens, food products, and medicinal preparations [36, 38, 54, 55]. Recently, a number of benchtop sequencers, such as Roche 454 GS Junior System, Ion Proton System, and Illumina MiSeq and MiniSeq, have been developed for routine tests in the laboratory [34, 36, 38, 56]. As NGS may produce sequencing errors, quality filtering and trimming of raw reads must be performed to remove erroneous data before barcoding analysis.

### 3.5. Reference Database

The accurate identification of animal species depends on the availability of reference sequence data, which are currently deposited in many public libraries, including GenBank, BOLD, Medicinal Materials DNA Barcode Database (MMDBD), International Nucleotide Sequence Database Collaboration (INSDC), and Barcode Index Number System (BIN). These databases contain a variety of sequences assigned to corresponding taxa, which is useful for comparative analysis of sequence variations. For example, GenBank, a commonly used database in barcoding studies, has included more than 1 Terabase sequence data with relatively broad taxon coverage [115]. Another database BOLD has already collected more than 2 million COI sequences from about 170,000 species, and INSDC has recorded extensive Cytb and ITS sequence information [38]. In addition, MMDBD focuses on the barcode information of medicinal plants and animals (over 1,700 species) listed in the Chinese Pharmacopoeia, American Herbal Pharmacopoeia, and other related references. Interestingly, MMDBD also includes sequence information of common adulterants and substitutes [116].

### 3.6. Sequence Analysis

Several comparative methods, including similarity-, distance-, and tree-based approaches, have been widely used to analyze sequence variations [38, 117]. The Basic Local Alignment Search Tool (BLAST) is a similarity-based algorithm, which matches the query sequence to those in reference databases and then provides a similarity score according to the portion of the query aligned to the reference [118]. For distance-based analysis, Kimura 2-parameter (K2P) model can be used to calculate the intraspecific and interspecific genetic distances among sequences, and then the barcoding gap can be used for species delimitation [117, 119]. In addition, tree-based methods are often used to establish the phylogenetic relationships, which assign query sequences to species on the basis of their membership of clusters in a barcode tree. The closest relative animal species will appear in a cluster, while distinct species should form discreet clusters. Several hierarchical clustering algorithms, including neighbor joining (NJ), maximum likelihood (ML), maximum parsimony (MP), and Bayesian inference (BI), have been used to establish phylogenetic tree, and a combination of these algorithms can provide more reliable identification as compared with a single algorithm [22, 117, 120, 121]. A number of commercial tools, such as MEGA, PHYLIP, and PAUP, can be used for tree construction and visualization.

## 4. Authentication of Animal-Based Traditional Medicine by DNA Barcoding

It is estimated that the animals used in TM are mainly from several phyla, including Chordata, Arthropoda, Echinodermata, Annelida, Mollusca, and Coelenterata. The authentication of genetic composition is important for quality control of animal-based TM and trade regulation of threatened medicinal animals. Recently, DNA barcoding as well as minibarcoding and metabarcoding has been successfully applied to identify single (Table 2) and multiple species (Table 3) in various types of animal-based TMs and discriminate the authentics from the adulterants [34, 36, 57–114].

### 4.1. Identification of Single Species

#### 4.1.1. Phylum Chordata-Derived Traditional Medicine

The phylum Chordata used in TM mainly includes mammals, reptiles, fishes, and amphibians. Among these vertebrates, many kinds of wild mammals as well as their organs and tissues, such as horns, scales, muscles, and gallbladders, have long been an important source of TM materials in many countries, especially those in Asia and Africa [2, 122]. For example, the horns from a migratory ungulate* Saiga tatarica* have been used in TCM for thousands of years, but the wild population of* S. tatarica* has rapidly declined in recent decades due to persistent hunting [57, 58]. CITES has listed this antelope in Appendix II since 1995, and the market trade of* Saiga* horns has been rigorously monitored. However, the horns from other species such as* Capra hircus *and* Procapra gutturosa* have been sold as* Saiga* horns in the market [57]. It is difficult to discriminate these horns as they share similar appearance, especially when they are processed in slices or powders. To distinguish the authentics from the adulterants, Chen et al. [57] recovered a 644 bp region of COI gene from well-preserved horns with specific primers and a 349 bp fragment from degraded horn samples with nested primers. Further analysis using K2P model and NJ tree method revealed that the mean intraspecific genetic distances of both full-length barcode and minibarcode are far less than the mean interspecific distances, and* S. tatarica* and its adulterant species can form independent clades in phylogenetic tree. Another famous horn-based TCM is Pilos antler, which is used for many illnesses including impotence, arthritis, and anemia [123]. It is officially derived from velvet antlers of* Cervus nippon* and* Cervus elaphus* according to the Chinese Pharmacopoeia [10], while the antlers of other deer such as* Rangifer tarandus *and* Dama dama* are often sold as the genuine products [62]. Recently, several studies have shown that using a set of DNA barcodes, including COI, Cytb, and 16S rRNA, can identify the biological origins of various animal horn samples and discriminate Pilos antler from its adulterants with high sensitivity [58, 62, 64]. Together, these results demonstrate that DNA barcoding and minibarcoding are capable of authenticating horn-based TM. Moreover, barcoding techniques have been used to analyze TM derived from other mammalian tissues, such as pangolin scales, deer musk, bear bile, and donkey-hide gelatin [36, 65–70]. For instance, pangolin scales, a rare TM used for many conditions including asthma and rheumatism, are derived from* Manis pentadactyla* according to the Chinese Pharmacopoeia [10]. However, the scales of other pangolin species often cause market confusion due to the scarcity of effective detection methods, and the illegal pangolin trade has escalated globally in recent years despite the legislation that all pangolin species have been listed in CITES Appendix II since 1994. To identify several batches of confiscated pangolin remains in Philippines and Hong Kong, several studies used COI and Cytb barcodes and found that barcoding approach can accurately assign unknown scales to specific species and distinguish different pangolin species [66, 67], indicating that DNA barcoding is a useful strategy for customs to suppress illegal trade of threatened mammals.

It is reported that many reptiles, including snakes, geckos, and turtles, play an important role in traditional folk medicine worldwide, and these reptile-based TMs have various therapeutic benefits, such as anti-inflammatory, sedative, and analgesic effects [124]. To identify the medicinal reptiles that exhibit similar morphology interspecifically, DNA barcoding provides a simple but reliable strategy (Table 2). For example,* Zaocys dhumnades* and* Bungarus multicinctus* are two important snakes used in TCM, and their dried bodies without the viscera have been widely applied to treat several disorders including rheumatoid arthritis, stroke, and convulsion [73–77]. However, many other snake species are marked as* Z. dhumnades* and* B. multicinctus* in the market, and the accurate identification of these snake-based TMs highly relies on professional experience. Interestingly, several recent studies have used a panel of full-length barcodes and minibarcodes including COI, 12S rRNA, 16S rRNA, and Cytb to analyze various snake specimens and related TMs collected from the wild and markets in China. The results showed that each sample can be identified as specific snake species, and* Z. dhumnades* and* B. multicinctus *as well as their adulterants can be clearly distinguished at the species level [73–77], indicating the efficacy of DNA barcoding and minibarcoding in authentication of snake-based TM. Another example is the dried body of* Gekko gecko*, which is traditionally used for relieving coughing and asthma [72]. Using 150-bp and 648-bp fragments of COI sequence,* G. gecko* specimens and their adulterants such as* G. japonicas* and* Calotes versicolor* have been found to exhibit significant barcoding gaps [72], further demonstrating the capacity of barcoding techniques in analyzing reptile-based TM.

As an indispensable group of vertebrates in aquatic animals, fish provides an enormous resource for humans as foods and medicines. Modern research has revealed that many fish species, especially marine fish, contain various substances with nutritional and pharmacological benefits [125]. For example, shark fins are not only the main constituent of the delicacy shark fin soup in Asian cuisines but also the precious TCM materials used for arthritis treatment and immune enhancement [97, 126]. Although twelve shark species have been listed in Appendix II of CITES, overfishing and illegal trade continue to induce a rapid decline of shark populations. In response to this issue, DNA barcoding has recently been adopted to authenticate shark species in the market [94–98]. For processed shark products, degraded genomic DNA may lead to unsuccessful amplification of full-length barcodes. Therefore, several studies used shorter fragments of COI (less than 200 bp) and showed that these minibarcodes can accurately identify CITES-listed shark species from processed fins and even fin soup and skin-care products [97, 98], demonstrating the capability of DNA minibarcoding in analyzing processed animal tissues. Another example is* Hippocampus* spp., which has a unique appearance in the family Syngnathidae and is a famous TM material used to treat impotence, asthma, and insomnia [84, 85]. As a mass of seahorses have been harvested and traded annually, all seahorse species have been included in Appendix II of CITES since 2004. To investigate the usage of seahorses in Chinese TM market, several recent studies employed DNA barcoding to analyze dried seashore specimens and revealed that both COI and Cytb barcodes can efficiently authenticate seahorse species and identify endangered species [84, 85]. Together, these studies demonstrate the availability of DNA barcoding in trade supervision of threatened fish species used in TM.

Amphibians are a group of ectothermic vertebrates characterized by their ability to exploit both aquatic and terrestrial habitats. Some of them, such as toads, frogs, and salamanders, are traditionally used to treat a number of ailments in many countries, including China, Japan, South Korea, and Spanish [127, 128]. For example,* Rana temporaria* and* Bufo gargarizans* are two common amphibians used in TM. The dried body and oil derived from* R. temporaria *are traditionally used to relieve cough and asthma, while the toad cake and skin of* B. gargarizans* have been used for treating heart diseases, skin ailments, and other systemic illnesses [10, 129]. However, other frog and toad species are often counterfeited as the authentics in the market. Recently, several studies have shown that distinct frogs and toads including* R. temporaria* and* B. gargarizans* can be clearly distinguished using DNA barcoding approach based on COI and 16S rRNA sequences [99, 100], which provides a reference for authentication of amphibian-based TM.

#### 4.1.2. Other Phyla-Derived Traditional Medicine

A variety of animal species in the phylum Arthropoda have been reported to be used in traditional therapeutic practices worldwide [130]. For example, the dried larvae of* Chrysomya megacephala* are traditionally used to treat malnutrition and skin and soft tissue wounds [101]. To discriminate this blowfly from other Diptera, a recent study used a full-length COI-based DNA barcoding and found that* C. megacephala* exhibited a complete nonoverlapping barcode divergence with other flies including the closest relative species* C. pinguis* [101], demonstrating the ability of DNA barcoding in authentication of insect-based TM. Moreover, barcoding technique has been applied to identify other medicinal animals in the phylum Arthropoda, such as scorpions and crabs [73, 106]. Interestingly, some animals have symbiotic associations with fungi, and they can be used together as TM materials. For the authentication of fungal species in such TMs, it is noteworthy that COI barcode cannot provide sufficient discrimination, while ITS barcode exhibits significant interspecific divergence [131]. For instance,* Cordyceps sinensis*, a well-documented tonic TCM widely used in Asia, is a combination of an entomopathogenic fungus and the larvae of the Hepialidae such as* Hepialus armoricanus* [104]. A number of close relative species such as* C. gunnii* and* C. cicadae* are commonly sold as* C. sinensis*, which seriously reduces the therapeutic efficacy or even leads to poisoning. To distinguish* C. sinensis* from counterfeit species, several recent studies have designed species-specific primers to amplify certain segments of ITS sequence and found significant barcoding gaps among these* Cordyceps *species [102–104]. Therefore, a combination of COI and ITS barcodes can be feasible to detect TM containing both insects and fungi. In addition to Arthropoda, a number of species in other phyla, including Echinodermata, Annelida, Mollusca, and Coelenterata, also provide important medicinal resources for traditional therapeutics [132–135]. Although DNA barcoding is relatively less addressed in TMs derived from these phyla, limited evidence has shown that it is still capable of identifying several species, including sea cucumbers, earthworms, oysters, and corals [108–113]. Together, these studies demonstrate that DNA barcoding and minibarcoding are accurate and efficient approaches to authenticate TM derived from a wide range of animal species.

### 4.2. Identification of Multiple Species

Recent concerns about the safety and legality of TM have prompted more rigorous surveillance. Interestingly, several studies have shown that DNA metabarcoding is capable of authenticating labeled species and detecting undeclared taxa in animal-based TM formulae (Table 3). For example, using 16S rRNA barcode combined with Roche GS Junior sequencing, half of the TCM preparations legally purchased in South Australia were found to contain DNA from undeclared animal or plant taxa [114]. Another recent study has developed a multilocus metabarcoding approach that employs 12 DNA barcode markers and Illumina MiSeq amplicon sequencing and revealed that Ma pak leung sea-dog hard capsules and Cobra performance enhancer hard capsules, both of which are used to treat sexual weakness, contain DNA from nondeclared taxa such as* Bos taurus *and* Homo sapiens* instead of labeled species [34]. Together, these studies suggest that metabarcoding can provide a pharmacovigilance measure for pre- and postmarket auditing of TM. In addition, metabarcoding has recently been used to identify threatened animal species in a variety of complex samples including TM preparations (Table 3). For instance, Coghlan et al. [114] have shown that 16S rRNA-based DNA metabarcoding can detect DNA from endangered animals* Panthera uncia* and possibly* Panthera tigris* in a TCM sample used to treat arthritis and pain. Moreover, similar technique was used to audit the genetic composition of some TCM samples seized by Australian Customs and Border Protection Service, and the results showed that* Saiga* antelope horn powder contained DNA from the known CITES-listed species* Saiga tatarica *and other species including goat and sheep, and Chu pak hou tsao san powder contained DNA from* Ursus thibetanus*, which is recorded in both CITES Appendix I and IUCN Red List [36]. These results suggest that DNA metabarcoding is useful for custom authority to analyze forensic specimens. Interestingly, DNA metabarcoding has also been used to identify the diet composition from animal secretions, such as the floral species of honey [136], further demonstrating its ability in biodiversity analysis.

## 5. Limitation and Prospect

Although DNA barcoding is an effective complement to conventional identification methods, it still has a few shortcomings [42, 137]; for example, some animal tissues such as horns, shells, and scales contain relatively small amount of DNA, resulting in difficulties for template amplification. Moreover, a false identification may be generated due to the contamination in DNA extraction and PCR reaction processes. Thus it is important to sample the tissues containing more cells and make sure all procedures are standardized. For example, to obtain a high yield and quality of DNA from animal horns, 75% ethanol can be used for sterilization, and middle layer between the bone core and outer sheath can be collected and then ground into powder in liquid nitrogen [57]. On the other hand, some TM preparations such as the extracts are highly processed, inducing degradation of DNA into very small fragments or even complete removal of DNA. In such cases, it is preferred to perform DNA barcoding analysis before the raw materials are processed. Another concern for complex samples with degraded DNA is that the amplification success of a barcode region may be different for distinct species due to varied gene copy number [20, 31]. It is thus necessary to employ multilocus barcoding and minibarcoding approaches and design novel primers for certain taxa.

The feasibility and accuracy of DNA barcoding are closely associated with both reference sequence data and taxonomically confirmed specimens. Although GenBank has included extensive COI sequences in a wide range of animal species, the information of some species used in TM is still lacking, and the sequence inventory of other barcodes such as 16S rRNA and Cytb also needs to be extended [38, 42]. Moreover, it is important to improve the availability of professionally authentic vouchers in public DNA databases as the misidentified species will generate incorrect sequence information [138, 139]. In addition, although MMDBD includes thousands of TM materials, the listed animal species and their adulterants are still insufficient, and the animal species used in other TM systems are also lacking. Therefore, more related databases should be established to provide sufficient bases for DNA barcoding analysis of animal-derived TM.

The quality of animal-based TM is known to be related to multiple factors, such as the specified tissues, the effective substances and even the growth stage. DNA-based analysis is feasible for genetic authenticity but unable to evaluate the pharmacological effects of TMs [19]; for example, DNA barcoding cannot distinguish different tissues from the same animal or determine the content of bioactive substances. It also fails to detect animal growth stages while some TM materials require animals at specific stages. Moreover, DNA barcoding is not feasible to identify the adulterants that do not contain DNA. To overcome these restrictions, it is necessary to combine genetic techniques with conventional approaches such as chromatography and metabolomics. Interestingly, a recent study used multidisciplinary techniques, including NGS, high performance liquid chromatography, and mass spectrometer, and showed that some TCM samples not only contained unlabelled animal species but also had undeclared pharmaceutical agents and excess heavy metals [114], indicating the potential of comprehensive analysis system in evaluating TM quality and reducing market fraud.

## 6. Conclusion

DNA barcoding offers a reliable and efficient strategy for the identification of authentic animal species and their adulterants in TM. It is noteworthy that the success of DNA barcoding is related to many factors, such as high quality of DNA and appropriate barcodes. For processed animal tissues with degraded DNA, minibarcodes usually exhibit higher success rate in species identification as compared with full-length barcodes. For complex mixtures such as TM formulae, metabarcoding provides a feasible approach to simultaneously detect multiple animal ingredients. With a global accumulation of open access reference sequences, DNA barcoding gradually becomes an authoritative approach in TM authentication. Despite these advantages, DNA barcoding still has several inherent limitations, such as inability to identify medicinal parts or determine compounds with pharmacological activities. Therefore, establishing a comprehensive identification system including barcoding and other techniques will provide more information for quality assessment and trade monitor of animal-based TM.

## Figures and Tables

**Table 1:** The commercial kits used for the extraction of DNA from different types of animal tissues.

Commercial kit	Purification method	Supplier	Starting material	Advantage
Qiagen DNeasy Blood and Tissue Kit	Silica-based technology	QIAGEN	Animal tissues and blood	Optimized protocols for a range of tissues, 96-well high-throughput formats

QIAamp DNA Stool Mini Kit	Silica-based technology	QIAGEN	Fresh or frozen stool	No organic extraction or alcohol precipitation, complete removal of contaminants and inhibitors

TIANamp Marine Animals DNA Extraction Kit	Silica-based technology	TIANGEN Biotech	Tissues of fish, shrimp, shellfish, crab, etc.	Specially developed for marine animals, no organic extraction

NucleoSpin® DNA RapidLyse Kit	Silica-based technology	Macherey-Nagel	Fresh, frozen, dried or ethanol preserved animal organs, eukaryotic cells, tail and ear clippings	Powerful lysis to efficiently release genomic DNA, superior genomic DNA yields

NucleoSpin® DNA Insect	Silica-based technology and NucleoSpin® Bead Tubes Type D	Macherey-Nagel	Fresh, frozen, dried or ethanol preserved insect or crustacean	NucleoSpin® Bead Tubes for efficient lysis of an exoskeleton

NucleoSpin® DNA Lipid Tissue	Silica-based technology and NucleoSpin® Bead Tubes Type D	Macherey-Nagel	Fresh or frozen, lipid-rich tissue (e.g., brain, adipose tissue, fatty fish tissue)	Special buffer for efficient removal of lipids, NucleoSpin® Bead Tubes for efficient lysis

Non-organic DNA Extraction Kit	Salting out precipitation	Merck millipore	Whole blood, body fluid, bone marrow, mononuclear cells, solid tissues	A simple and non-toxic way to isolate high molecular weight genomic DNA

OmniPrep™ kit	Unique precipitation reagents	G-Biosciences	Tissues from different species including animal, plant, bacteria, yeast and fungus	High yield of ~100 kb genomic DNA, no organic extraction

MagAttract Blood DNA/RNA Kit	Magnetic bead-based technology	MoBio	Fresh whole blood, frozen whole blood, buffy coat, plasma	Works with all blood types, novel ClearMag magnetic particle technology

ChargeSwitch® gDNA Mini Tissue Kit	Magnetic bead-based technology	Invitrogen	Animal tissues such as tail, spleen, bone and hair	High-purity DNA extraction, improved performance with accompanying magnetic rack

MagListo™ 5 M Forensic Sample DNA Extraction Kit	Magnetic bead-based technology	Bioneer	Whole blood, saliva, urine, hair, finger nail, bone	Suitable for forensic samples, a rapid and cost-effective DNA extraction

**Table 2:** Identification and authentication of animal species in traditional medicine by DNA barcoding and minibarcoding.

Medicinal species	Medicinal part	Therapeutic benefit	Adulterant species	Tested sample	Tested barcode	Reference
*Chordata: Mammalia*						

*Saiga tatarica* ^*∗*#^	Horns	Treat fever, headaches, eye ailments, convulsion, epilepsy and agitation	*Capra hircus*, *Ovis aries*, *Procapra gutturosa*, and other ungulate species	Horns, muscles, skin	349 bp, 487 bp and 644 bp of COI	Chen et al. [57] Yan et al. [58] Cao et al. [59]

*Bubalus bubalis*	Horns	Treat fever, delirium, hemoptysis and convulsion	*Bos grunniens*,* Bos taurus domesticus*	Horns	487 bp of COI	Yan et al. [58]

*Ceratotherium simum* ^*∗*^, *Diceros bicornis*^*∗*#^, *Rhinoceros unicornis*^*∗*^, and other rhino species^*∗*^	Horns	Treat fever, influenza, convulsion, delirium, hemoptysis and abscess	*Bubalus bubalis*, *Bos taurus*, *Equus ferus*^*∗*#^	Horns, bones, hair, blood, feces, skin	230 bp of Cytb	Ewart et al. [60]

*Cervus elaphus* ^*∗*^ *, Cervus nippon*	Velvet antlers, tendons	Treat impotence, arthritis, anemia, knee weakness, skin ailments and urinary disorders, improve muscle tone and nerve function	*Elaphurus davidianus* ^#^, *Dama dama*^*∗*^, *Rangifer tarandus*, *Bos *spp., *Bubalus bubalis*	Antlers, tendons	COI, Cytb, 12S rRNA, 16S rRNA	Yan et al. [58] Chung et al. [61] Sin et al. [62] Jiang et al. [63] Cai et al. [64]

*Moschus berezovskii* ^*∗*#^	Musk	Treat stroke, coma, convulsion and sores		Skin, blood, muscles	627 bp of COI, 723 bp of *D*-loop	Yang et al. [65]

*Manis pentadactyla* ^*∗*#^	Scales	Treat asthma, rheumatism, arthritis and stomach ulcers, enhance microcirculation	*Manis culionensis* ^*∗*#^, *Manis javanica*^*∗*#^, *Manis tricuspis*^*∗*^, and other pangolin species^*∗*^	Scales, muscles, blood	~650 bp of COI, 400 bp of Cytb, 576 bp of *D*-loop	Luczon et al. [66] Mwale et al. [67]

*Equus asinus*	Gelatin	Promote hematopoiesis and arrest bleeding	*Equus caballus*,* Bos taurus*, *Sus scrofa*	Glue, hair, muscles	<100 bp of Cytb	Kumeta et al. [68]

*Ursus thibetanus* ^*∗*^	Bile, gallbladders	Treat fever, epilepsy and haemorrhoids, alleviate inflammation and pain	*Ursus americanus* ^*∗*^	Bile, coats	COI, Cytb, 16S rRNA	Coghlan et al. [36] Janjua et al. [69] Peppin et al. [70]

*Panthera tigris* ^*∗*#^	Bones	Relieve pain and convulsion	*Canis lupus familiaris*	Tanned leathers	Cytb	Jun et al. [71]

*Lutra sumatrana* ^*∗*#^	Skin	Treat fever		Tanned skin	COI	Janjua et al. [69]

*Chordata: Reptilia*						

*Gekko gecko*	Dried body without viscera	Treat cough, cold, impotence, asthma and tuberculosis	*Calots versicolor*,* Gekko japonicas*, etc.	Muscles, livers	648 bp and 150 bp of COI	Gu et al. [72]

*Zaocys dhumnades*, *Bungarus multicinctus*	Dried body without viscera	Treat sores, abscesses, eye infection, rheumatoid arthritis and stroke, relieve pain, spasm and convulsion	*Agkistrodon acutus*, *Coelognathus radiates*,* Cyclophiops major*, *Dinodon rufozonatum*,* Elaphe carinata*, and other snake species	Muscles, other tissues, concentrated granules	48–658 bp of COI, 142–460 bp of 16S rRNA, 183–308 bp of Cytb, 83–350 bp of 12S rRNA	Jiang et al. [73] Cao et al. [74] Wang et al. [75] Li et al. [76] Chao et al. [77]

*Chelonia mydas* ^*∗*#^	Shell	Treat vertigo, agitation and insomnia		Blood, tissues	815 bp of COI	Naro-Maciel et al. [78]

*Pelodiscus sinensis*	Shell	Treat vertigo, agitation and insomnia	*Dogania subplana* ^*∗*^, *Lissemys punctata*^*∗*^, other turtle species	Blood, tissues	621 bp and 650 bp of COI	Kundu et al. [79] Reid et al. [80]

*Caiman crocodilus* ^*∗*^, *Gavialis gangeticus*^*∗*#^, and other crocodile species^*∗*^	Muscles	Treat rheumatism, asthma, epilepsy and stroke		Blood, tissues	645 bp and 750 bp of COI, 1290 bp of 16S rRNA	Meganathan et al. [81] Jogayya et al. [82] Eaton et al. [83]

*Chordata: Actinopterygii*						

*Hippocampus *spp.^*∗*^	Dried body without viscera	Treat impotence, asthma, insomnia, infections and sores		Fins	653 bp of COI, 1140 bp of Cytb	Chang et al. [84] Hou et al. [85]

*Syngnathoides biaculeatus*,* Solegnathus hardwickii*, *Syngnathus acus*	Dried body	Treat impotence, incontinence, asthma and arteriosclerosis	*Doryichthys boaja*, *Hippichthys cyanospilus*,* Pegasus volitans*, and other pipefish species	Muscles, fins	649 bp of COI, ~385 bp of 12S rRNA	Zhang et al. [86] Gao et al. [87]

*Epinephelus *spp.	Muscles	Alleviate hypertension and hyperglycemia		Fins, livers, muscles	~700 bp of COI	Torres et al. [88]

*Oncorhynchus *spp.,* Salmo *spp.	Muscles	Alleviate hypertension and hyperglycemia		Tissues	109–650 bp of COI	Rasmussen et al. [89]

*Scomber* spp.	Muscles	Relieve fatigue and nervosism		Muscles	226 bp of Cytb	Botti and Giuffra [90]

*Decapterus Maruadsi*	Muscles	Treat dysentery and hemoptysis		Tissues	652 bp of COI	Mat Jaafar et al. [91]

*Chordata: Chondrichthyes*						

*Manta alfredi* ^*∗*^, *Manta birostris*^*∗*^,* Mobula japonica*^*∗*^,* Mobula kuhlii*^*∗*^, *Mobula tarapacana*^*∗*^, and other mobulid ray species^*∗*^	Gill rakers	Relieve arthritis, asthma, children measles and skin sores and boils		Gill rakers	652 bp and 761 bp of COI, ~500 bp of 16S rRNA, 1033 bp of NADH2	Asis et al. [92] Zeng et al. [93] Steinke et al. [94]

*Alopias vulpinus* ^*∗*^,* Alopias pelagicus*^*∗*^, *Carcharhinus falciformis*^*∗*^, *Rhincodon typus*^*∗*#^, *Sphyrna zygaena*^*∗*^, and other shark species^*∗*^	Fins	Treat arthritis, improve immune system function		Fins, fin soup, muscles, liver oil, skin-care products	~110–650 bp of COI, ~500 bp of 16S rRNA	Steinke et al. [94] Liu et al. [95] Chuang et al. [96] Fields et al. [97] Cardeñosa et al. [98]

*Chordata: Amphibia*						

*Rana temporaria*	Dried body, oil	Treat throat inflammation, cough, asthma and arthritis		Tissues	16S rRNA	Maya-Soriano et al. [99]

*Bufo gargarizans*	Toad cake, skin	Treat heart diseases, skin ailments and some cancers		Toe tips, muscles, livers	565–602 bp of COI	Che et al. [100]

*Andrias davidianus* ^*∗*#^	Dried body, skin	Treat anemia, dysentery, chills and burns		Toe tips, muscles, livers	565–602 bp of COI	Che et al. [100]

*Batrachuperus pinchonii*	Dried body, skin	Treat rheumatism and stomachache		Toe tips, muscles, livers	565–602 bp of COI	Che et al. [100]

*Arthropoda: Insecta*						

*Chrysomya megacephala*	Dried larvae	Treat malnutrition and skin and soft tissue wounds	*Lucilia illustris*, *Musca sorbens*, and other fly species	Hind legs	658 bp of COI	Qiu et al. [101]

*Cordyceps sinensis*	Hepialidae caterpillars and fungal stromata	Treat asthma, bronchitis, erectile dysfunction, diabetes, cough, cold and jaundice	*Cordyceps gunnii*, *Cordyceps gracilis*, *Cordyceps hawkesii*, and other *Cordyceps *species	Stromata	146–493 bp of ITS	Xiang et al. [102] Lam et al. [103] Liu et al. [104]

*Arthropoda: Arachnida*						

*Mesobuthus martensii*	Dried body, venom	Treat rheumatoid arthritis, stroke, epilepsy, multiple sclerosis, chronic pains and some cancers	*Mesobuthus gibbosus*, *Mesobuthus eupeus*	Tissues, concentrated granules	48–94 bp of COI, 83–350 bp of 12S rRNA, 142–460 bp of 16S rRNA	Jiang et al. [73] Ortiz et al. [105]

*Arthropoda: Malacostraca*						

*Charybdis lucifera*, *Portunus pelagicus*, *Scylla serrata*, and other crab species	Shell	Treat cancer, inflammatory bowel disease and chilblain		Muscles	650 bp of COI	Vartak et al. [106] Mirzapur et al. [107]

*Echinodermata: Holothuroidea*						

Holothurian species	Whole body	Treat joint pain, arthritis, tendonitis and sprain		Tube feet, arms, gonads	528 bp and 657 bp of COI	Uthicke et al. [108] Ward et al. [109]

*Annelida: Clitellata*						

*Eisenia fetida*	Dried body	Treat fever and convulsion		Tissues	581 bp of COI	Rombke et al. [110]

*Mollusca: Bivalvia*						

*Crassostrea rivularis, Crassostrea gigas*	Shell	Treat impotence, insomnia, epilepsy, bone loss and ulcer	*Hyotissa hyotis*,* Ostrea edulis*, and other oyster species	Muscles	COI, 16S rDNA	Hsiao et al. [111] Liu et al. [112]

*Cnidaria: Anthozoa*						

*Corallium rubrum*	Skeleton	Treat arthritis and ulcer	Other coral species	Fragments	1597 bp of COI	Uda et al. [113]

^*∗*^Included in the Appendices of the Convention on International Trade in Endangered Species (CITES); ^#^Recorded as endangered, critically endangered or extinct in the wild status in the International Union for Conservation of Nature (IUCN) Red List of Threatened Species.

**Table 3:** Authentication of animal species in traditional medicine by DNA metabarcoding.

Tested sample	Therapeutic benefit	Barcode	Sequencing method	Labeled animal species	Identified animal species	Reference
Ma pak leung sea-dog hard capsules	Enhance sexual and physical function	12 barcodes	Illumina MiSeq amplicon	*Cervus* sp.	*Bos taurus*	Arulandhu et al. [34]

Cobra performance enhancer hard capsules	Enhance sexual and physical function	12 barcodes	Illumina MiSeq amplicon		*Bos taurus*, *Homo sapiens*	Arulandhu et al. [34]

Mongnan Tianbao Pills	Enhance sexual and physical function	16S rRNA	Roche GS Junior	*Hippocampus* sp.^*∗*^, *Callorhinus* sp.	*Cervus elaphus* ^*∗*^, *Bos taurus*	Coghlan et al. [36]

Ling Yang Ge Gen Cold Remedy	Relieve fever, cold and influenza	16S rRNA	Roche GS Junior	*Saiga tatarica* ^*∗*#^	*Capra* sp., *Ovis* sp.	Coghlan et al. [36]

Laryngitis Pills	Treat throat sores, acute tonsillitis and mumps	16S rRNA	Roche GS Junior	*Bufo* sp.,* Bos taurus*, *Ursus* sp., *Moschus *sp.^*∗*#^, Bivalvia	*Bufo* sp., *Bubalus* sp.	Coghlan et al. [36]

*Saiga* Antelope Horn Powder	Treat fever, headaches, convulsions and epilepsy	16S rRNA	Roche GS Junior	*Saiga tatarica* ^*∗*#^	*Saiga tatarica* ^*∗*#^, *Capra* sp., *Ovis* sp.	Coghlan et al. [36]

Yatong Yili Wan	Relieve gingival pain	16S rRNA	Roche GS Junior	Cicadoidea, Bivalvia, *Sus* sp.	*Bufo* sp.	Coghlan et al. [36]

Powder in vials	Alleviate inflammation	16S rRNA	Roche GS Junior	*Ursus* sp.^*∗*^, *Anser* sp.	*Ursus thibetanus* ^*∗*^	Coghlan et al. [36]

Chu pak hou tsao san	Relieve cough, reduce phlegm	16S rRNA	Roche GS Junior	*Macaca *sp, Bivalvia,* Bos taurus*	*Ursus thibetanus* ^*∗*^	Coghlan et al. [36]

TCM 8	Treat arthritis and pain	16S rRNA	Roche GS Junior		*Canis *sp., *Panthera uncia*^*∗*^, *Panthera tigris*^*∗*#^	Coghlan et al. [114]

TCM 11		16S rRNA	Roche GS Junior		*Canis *sp., *Rattus* sp., *Deinagkistrodon* sp.	Coghlan et al. [114]

TCM 20, TCM 26		16S rRNA	Roche GS Junior		*Bos *sp.	Coghlan et al. [114]

TCM 25		16S rRNA	Roche GS Junior		*Felis *sp., *Rattus* sp.	Coghlan et al. [114]

^*∗*^Included in the Appendices of the Convention on International Trade in Endangered Species (CITES); ^#^Recorded as endangered, critically endangered or extinct in the wild status in the International Union for Conservation of Nature (IUCN) Red List of Threatened Species.
